# Five Reasons for Using Heavy Gold Foil in Filling Teeth

**Published:** 1888-02

**Authors:** E. G. Betty


### THE DENTAL REGISTER.

VOL. XLII.] FEBRUARY, 1888. [No. 2.

### COMMUNICATIONS.

### Five Reasons for Using Heavy Gold Foil in the Filling of Teeth.

BY E. G. BETTY, D.D.S.

[Read before the Odontological Society of Cincinnati.]

*Mr. President and Fellow Members:*—

I was requested to give in a succinct form, five reasons for using heavy foil. To this I replied that I had nothing new to offer that would be of interest and that the matter has already been frequently and thoroughly canvassed. When assured, however, that it was for the purpose of eliciting a discussion, I consented to thus briefly bring to your notice a few of the salient points in a proceeding that I am generally known to champion. With your permission, however, I shall not confine myself strictly to the title of this sketch but consider the subject as a whole, bringing in the reasons as they happen to fall into place.

The whole subject of the use of gold in filling teeth, is, without doubt, the most important one the profession has had to deal with and by common consent it is the solution of the problem, how best to preserve the teeth. Its paramount importance has stimulated nearly every one to write or say something about it and for this reason the literature of the topic is of almost endless extent and variety. Conceding that gold is *the material* above all others, the next question that arises is the form in which it is best adapted to secure the common object, *viz:* the saving of the tooth. In this respect scarcely two persons agree, every one advancing some reason or other why this, that, or the other form is the one which offers the most advantage, and I may say right here that, all forms, be they what they will, possess some one or more of the many requisites and which in some instances and under peculiar circumstances commend themselves to the approbation of the operator. Laying this aside, however, there are some properties of gold as a metal which all forms possess and which can not be modified by any known method of manipulation ; I refer to color, density, specific gravity, thermal and electric conductivity. These properties then, we must leave out of the account and proceed to those which may in some degree be changed to suit the requirements. Of these qualities these are welding, malleability and ductility, all of great importance to the successful manipulation of the metal in the act of filling, however, not near so much so as the first.

Another quality that gold possesses above all other material used for filling, is indestructibility ; no reagents yet found in the mouth under ordinary conditions being able to more than discolor it, and even that is likely due to the presence of some extraneous element incorporated in it during its preparation—usually iron. Hardness is another quality which gold possesses though in a limited degree, other substances used for filling greatly excelling it in this respect, though for all the purposes of a practical filling it is sufficient.

I have now, gentlemen, given you in brief the essential properties needed in a filling material and gold possesses all of those mentioned. It is now necessary for us to determine which *form* of gold, as prepared for the dentist, possesses these qualifications to the greatest extent and degree.

So far as my individual opinion is concerned, I believe that heavy gold foil is *the form* which embraces the most, and by enumerating them, I will both answer the five reasons and conclude my remarks. I may as well say here that No. 60 is the weight best evidencing the requisite qualities of gold as a filling material, together with ease of manipulation in the introduction of the filling.

It enables the operator to introduce the maximum amount of material with the minimum amount of space occupied in the introduction, a point you will all readily appreciate.

The welding property in the cold state is best manifested when the amount of material in use at one time is sufficiently thick to withstand the percussion of the mallet, the serrations of the plugger being unable to cut through and consequently comminute the layers as they are welded together. The fewer the surfaces brought together in a mass such as a filling is, the stronger will be the cohesion of that mass by reason of there being fewer parts to divide the strain. That is a principle in mechanics that is well appreciated by the builder, the boiler-maker, and many others that might be mentioned.

In the wear and tear of a filling in the mouth, especially those operations we denominate "contour," heavy foil will stand longer and better with little or no tendency to chip or granulate. This is conceded by those who use pellets almost exclusively, they almost invariably advocating the "covering" of a filling of light numbers with a few layers of heavy foil. There are other points I might mention, among them mallets—but I have probably exceeded the time allotted me and it is preferable to hear them brought out in the discussion.

